# Vaping-Induced Sepsis and Rapidly Evolving Pleural Effusion in a Young, Otherwise Healthy Male

**DOI:** 10.7759/cureus.25327

**Published:** 2022-05-25

**Authors:** Bilal Malik, Atefeh Kalantary, Abhijeet Ghatol, Arvind Kunadi

**Affiliations:** 1 Internal Medicine, McLaren Health Care, Michigan State University, Flint, USA; 2 Internal Medicine, McLaren Flint, Michigan State University, Flint, USA; 3 Pulmonary and Critical Care Medicine, McLaren Health Care, Flint, USA; 4 Internal Medicine and Nephrology, McLaren Health Care, Michigan State University, Flint, USA

**Keywords:** electronic cigarettes 'e-cigarettes' vaping' e-smoking, pleural empyema, adolescent nicotine use, e-cigarette and vaping product use associated lung injury (evali), vaping

## Abstract

There have been increasing reports of electronic cigarette (e-cigarette) or vaping use-associated lung injury (EVALI), and the evolving literature demonstrates that the solvents used to dissolve flavors, including vitamin E, may be responsible, at least in part, for the injuries associated with this form of smoking. We present the case of a 26-year-old, otherwise healthy, African American male with a history of heavy use-cigarette use who presented with pleuritic chest pain. He was found to be septic and developed a rapidly accumulating pleural effusion with loculations during his hospitalization. He eventually underwent a thoracotomy due to his deteriorating respiratory status. This case highlights the importance of physician awareness regarding the growing evidence base suggesting that electronic cigarettes and vaporized tobacco products are not as safe as they have been perceived to be. Physicians should screen for and advise patients regarding the risks associated with the use of such products.

## Introduction

Over the last five years, the electronic cigarette (e-cigarette) and vape market have nearly tripled in value, being worth approximately 19.3 billion dollars at present [1]. The initial projection by the media portrayed a very positive outlook on e-cigarettes and vaporized tobacco alternatives, with the claim that there were minimal health effects as compared to cigarette smoking. Traditional smoking releases thousands of chemicals and toxins during the process of burning tobacco within cigarettes [2]. Alternatives, such as e-cigarettes, which may contain a lesser absolute number of chemicals and constituents, were initially thought to be healthier alternatives [2]. However, with the increasing body of literature regarding the toxicities associated with vaping, it is now becoming more evident that there are potential harms of vaping that are not so different from traditional tobacco combustion [1,2]. Importantly, the solvents used to dissolve flavors and active ingredients, including nicotine and tetrahydrocannabinol (THC), within “E-liquids” can vary based on intrinsic properties and potential effects [2]. Propylene glycol, vegetable glycerin, ethylene glycol, toluene, 1,3-propanediol, polyethylene glycol 400, medium-chain triglycerides, and vitamin E acetate may be utilized as solvents in these products [2]. We present the case of a 26-year-old, otherwise healthy, African American male with a history of heavy vaporized tobacco product use who presented with complaints of pleuritic chest pain, became septic, had a rapidly evolving pleural effusion with loculations, and ultimately required thoracotomy for resolution.

## Case presentation

Our patient was a 26-year-old African American male patient with a known history of heavy vaping but no significant past medical history, who initially presented to urgent care with complaints of pleuritic chest pain on his right side and associated dyspnea. Computed tomography (CT) scan of the chest performed in the urgent care setting demonstrated mildly enlarged right hilar lymph nodes, a small right-sided pleural effusion, and bibasilar airspace opacities, with right greater than left. He was then transferred to our institution for further workup and management. Upon arrival, the patient’s vital signs were stable with a blood pressure of 133/84, heart rate of 65 beats per minute, a temperature of 98.2 F, respiratory rate of 20 breaths per minute, and oxygen saturation of 96% on five liters per minute (LPM) nasal cannula (NC). His cardiovascular and respiratory system examinations were unremarkable aside from soft bi-basilar crackles. His initial laboratory studies were significant for a white blood cell count of 31 × 10^9^/L with an absolute neutrophil count of 27.3 × 10^9^/L, mild anemia with a hemoglobin of 13.0 g/dL, aspartate aminotransferase of 71 U/L, and alanine aminotransferase of 128 U/L (lab values summarized in Table 1). All remaining lab values, including lactic acid, troponins, and lipase, were within the normal range. The patient was admitted to the hospital for further observation and started on azithromycin and ceftriaxone for antimicrobial coverage.

**Table 1:** Summary of significant lab findings.

Lab study	Value (reference range)
White blood cells	31 × 10^9^/L (4-11 × 10^9^/L)
Absolute neutrophil count	27.3 × 10^9^/L (2.5-6 × 10^9^/L)
Hemoglobin	13.0 g/dL (14-18 g/dL)
Aspartate aminotransferase (AST)	71 U/L (10-40 U/L)
Alanine aminotransferase (ALT)	128 U/L (7-56 U/L)

**Figure 1:**
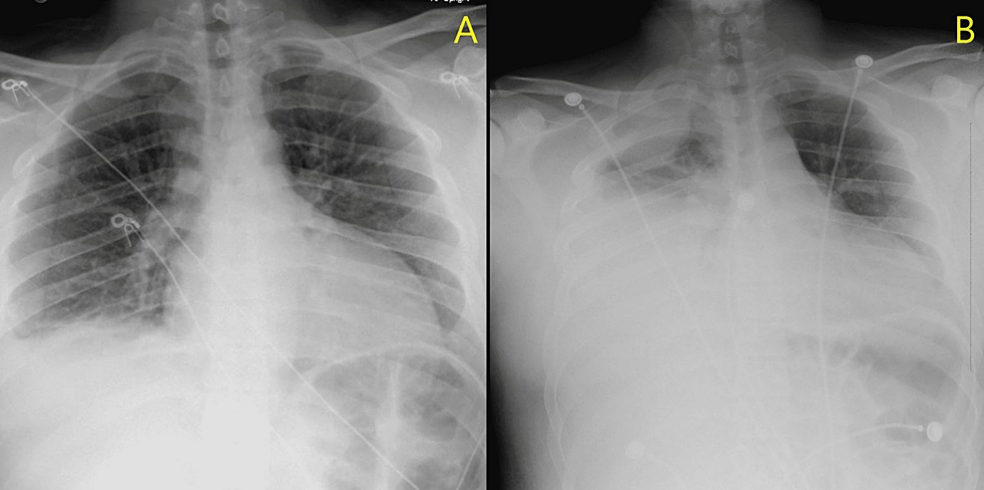
Chest x-ray images demonstrating rapid accumulation of a loculated pleural effusion. (A) Initial presentation and (B) three days later.

**Figure 2:**
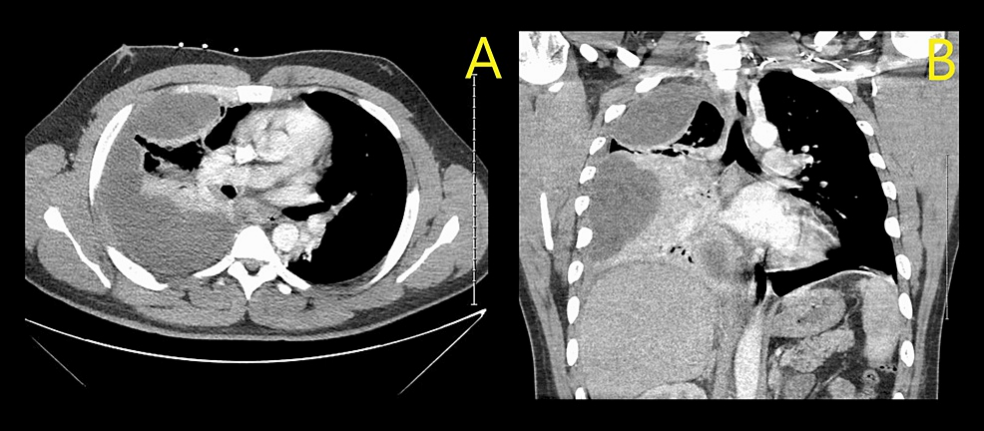
CT scans of the chest without contrast demonstrating loculated pleural effusion and atelectatic lung beneath it (A) transverse view and (B) coronal view.

Over the next 48 hours, the patient’s pain was managed with hydrocodone/acetaminophen and as-needed morphine for breakthrough pain. He was weaned off the nasal cannula during this time and encouraged to perform incentive spirometry. A follow-up chest x-ray (CXR) was ordered to evaluate the status of the right-sided pleural effusion. This CXR demonstrated progression into a large, right-sided pleural effusion (Figure 1A, 1B). The patient did develop fevers over the following night, reaching as high as 101.1 F. His vital signs at this time included a heart rate of 106 bpm, respiratory rate of 22 breaths per minute, blood pressure of 153/94, and oxygen saturation of 97% on 2 LPM NC. At this time, the National Early Warning Score (NEWS2) for acute illness was calculated, identifying our patient to be at medium risk for sepsis, and demanding an urgent response. His NEWS2 score was six (one point for pulse, one for temperature, two for respiratory rate, and two for oxygen use). Following his fever and increasing respiratory demands, a computed tomography (CT) scan of the chest without contrast was performed and was significant for a large, loculated right pleural effusion, complete right lower lobe collapse, the incomplete collapse of the right middle lobe, and multiple atelectatic areas within the right lung (Figure 2A, 2B).

Cardiothoracic surgery was consulted due to the rapidly evolving, loculated pleural effusion. Right thoracotomy, evacuation of the suspected empyema, and decortication of the right lung were performed. During the procedure, the patient was noted to have thick, gelatinous material upon entry into the pleural space. Similar gelatinous material was evacuated from around the right upper, right lower, and right middle lobes, and all material was cleared during the procedure. Of note, no frank pus was seen in the procedure. Due to the patient’s otherwise unremarkable history, the lack of an alternative plausible etiology (cardiac, rheumatologic, or neoplastic processes), and the lack of other predisposing factors for the development of such rapidly accumulating pleural effusion and sepsis, the patient was diagnosed with a probable case of e-cigarette or vaping use-associated lung injury (EVALI). He had been using vaporized tobacco products within the 90 days preceding presentation, had significant opacities on chest imaging, no plausible alternative etiology, and a high degree of clinical suspicion from the managing physicians.

Cultures were taken during the procedure and grew lactamase-negative *Bacteroides uniformis*. Prior to this, all cultures were negative, including blood, sputum, fungal, and anaerobic cultures. He successfully recovered post-op, the chest tubes were removed without complications, and he was sent home with a peripherally inserted central catheter (PICC) line on intravenous ertapenem for an additional two weeks after discharge. He was followed up with his primary care provider (PCP) in the outpatient setting and has successfully returned to work with no further complications following the completion of antimicrobial therapy.

## Discussion

The potential harms of the primary use of e-cigarettes have been a subject of increasing debate across various forms of media. Initially, e-cigarettes and vaporized tobacco alternatives were portrayed as very positive, with claims that there were minimal health effects as compared to cigarette smoking. This has given impetus to an increasing trend of replacement of standard cigarette smoking to e-cigarettes, especially among the younger population. A significant degree of marketing was conducted to advertise these alternatives as tobacco cessation tools [3]. A recent report from Public Health England reinforced prior recommendations that e-cigarettes are a safer alternative and that individuals should consider this as an alternative [4]. These claims have become problematic with a growing body of literature suggesting the contrary.

The growing number of health concerns that have arisen from vaping, which have collectively been termed “vaping product-associated lung illness (EVALI)” or “vape lung” [2]. As of February 18, 2020, the Centers for Disease Control and Prevention (CDC) had reported that a total of 2,807 people had been hospitalized with lung injuries or deaths because of e-cigarettes or vaping. Based on these statistics, the CDC proposed the diagnostic criterion for a “confirmed case” of EVALI as follows: (1) use of an e-cigarette or a related product (e.g., “vaping” or “dabbing”) in the previous 90 days; (2) lung opacities on the chest imaging (radiograph or CT scan); (3) exclusion of lung infection; and (4) absence of a likely alternative diagnosis (e.g., cardiac, neoplastic, and rheumatologic) [5]. In patients, such as our patient, who did not have a complete pulmonary infectious work-up (i.e., respiratory viral panel, influenza testing, etc.) but met the remaining criteria for EVALI, the CDC definitions label them as a “probable case” of EVALI. Alternatively, if the clinicians involved find themselves faced with a positive result on an infectious work-up but do not believe the presentation to be solely due to underlying infection, the patient may also be called a probable case of EVALI [6]. Ultimately, EVALI is considered a diagnosis of exclusion for which no direct confirmatory testing exists [6], necessitating clinicians to employ a high degree of clinical judgment based upon the evidence presented to them and the suggested definitions by the CDC as referenced above.

Public health investigations led to the identification of vitamin E acetate as the most likely etiologic agent in EVALI. Furthermore, research demonstrated that phosphatidylcholines (a component of surfactant) transition from a gel to a liquid crystalline phase when exposed to increasing amounts of tocopherols, such as vitamin E acetate. The transition to a liquid crystalline phase causes the surfactant to lose its ability to maintain the surface tension that is necessary to prevent collapse within the alveoli. This provides a potential explanation for how vitamin E acetate causes respiratory dysfunction in the lungs of patients experiencing EVALI. Our patient may have experienced a similar transition into a precipitate surrounding the lung tissue and causing a loculated empyema-like picture on imaging (Figure 1, 2). Unfortunately, during the time of treatment, the patient’s evacuated effusion specimen was not sent for pathology review and the constituents are unknown. It is only clear that there was no frank lobular pneumonia, that there was no frank pus during thoracotomy (excluding an empyema), and the rapid accumulation of the effusion caused the underlying lung to become largely atelectatic. In a New England Journal of Medicine (NEJM) study by Blount et al. on e-cigarette-related complications, including EVALI, bronchoalveolar lavage (BAL) samples were also tested for toxins. Again, our patient did not undergo bronchoscopy, and these data were not available for comparison against the cases in the study by Blount et al. [6].

Over time, the long-term use of these products has been identified to be related to the outbreak of acute vaping-associated lung injury in the USA [7]. Pneumonia is diagnosed in approximately one million hospitalized patients in the United States each year, with 20-40% of these patients also developing parapneumonic effusions concurrently. In cases of bilateral effusion, the mortality rate can increase by 6.5 times compared to patients hospitalized with only pneumonia. By definition, a parapneumonic effusion is considered any pleural effusion occurring secondary to pneumonia (bacterial or viral) or lung abscess. An abscess may be termed as an empyema when there is pus in the pleural space. A computed tomography (CT) scan of the chest is the diagnostic modality of choice. Several acute pulmonary diseases, including primary spontaneous pneumothorax and acute eosinophilic pneumonia, have also been reported as a consequence of e-cigarette usage [2]. Potential mechanisms of such injuries have been described in murine models whereby oxidative stress contributes to the development of pathology [8]. Due to the lack of long-term data on the safety of chronic vape/e-cigarette use, clinicians should be aware of the consequences of EVALI to effectively identify and manage these conditions and their complications.

Parapneumonic effusions are characterized based on the size, microbiology, and chemistry of the fluid (for example, type of bacteria present, lactate dehydrogenase (LDH) content, protein content, etc.). The lesion may be categorized as small when it measures less than 10 mm in thickness on a decubitus, free-flowing effusion. Generally, thoracentesis is not recommended in those with small effusions due to the risk of poor outcomes being very low, even without intervention. The second category is small to moderate effusions, which includes effusions that are 10 mm in thickness, less than one half of the hemithorax, free-flowing, negative gram stain and culture of the pleural fluid, pleural fluid glucose of more than 60 mg/dl, and a pleural fluid pH above 7.2. Thoracentesis is indicated in such patients, but the risk of poor outcomes remains low overall. The third category must fulfill at least one of the following criteria: the effusion of more than one-half of the hemithorax is loculated or associated with a thickened parietal pleura, has a positive gram stain or culture, a pleural fluid pH of less than 7.2, or pleural fluid glucose of less than 60 mg/dl. If a patient fulfills any of these criteria, they are deemed to be at moderate risk for poor outcomes. The worst outcomes and highest mortality have been associated with category four pleural effusions, which involve the presence of any pustulent material. Drainage is indicated alongside appropriate antimicrobial therapy in such cases.

It is important to note that, with the exception of category 1 pleural effusions, the majority of effusions should be managed with thoracentesis and subsequent fluid analysis. The classification of each respective disease category will dictate subsequent management decisions. The most common method of draining a parapneumonic effusion is tube thoracostomy [9]. In patients with loculated, complex fibrinopurulent parapneumonic empyema, the primary treatment modality of choice is video-assisted thoracic surgery (VATS). This method has demonstrated higher efficacy, a shorter duration of hospital stay, and more cost-effectiveness [10]. VATS is also used in patients with failed complete drainage of an empyema via a tube thoracostomy. The use of intrapleural fibrinolytic therapy should be reserved for patients in centers without access to VATS [9]. Initially, our patient was planned to have VATS, but upon entry into the pleural space, the presence of thick, gelatinous material prevented the use of this technique. Instead, our patient was evacuated by right thoracotomy at the discretion of the surgeon.

Several cases documenting the complications of vaping have been reported in the literature to date. For example, one such report featured a 38-year-old female who had a history of vaping and e-cigarette use, which was complicated by a left-sided empyema and managed with IV antibiotics and fibrinolytics [11]. In contrast to our case, this patient did grow *Staphylococcus aureus* and was managed solely with noninvasive methods. Another example featured a male patient in his 40s who presented with rapid deterioration requiring intubation and eventual extracorporeal membrane oxygenation support [1]. Despite our patient’s deteriorating respiratory status, he did not require such high levels of respiratory support and was managed on a nasal cannula for the majority of his admission. These cases, though similar to our patient’s scenario, demonstrate the possible range of complications from vaping.

## Conclusions

We presented the case of a 26-year-old African American male with a history of heavy vaporized tobacco product use who presented with complaints of pleuritic chest pain, was eventually found to have a rapidly evolving pleural effusion with loculations, and ultimately required thoracotomy for resolution. This case highlights the importance of educating both providers and patients regarding the dangers of vaporized products used as tobacco substitutes and alternatives. We recommend providing specific and focused interventions at each visit. When the patient is in the pre-contemplation stages of smoking cessation, they should be prompted each visit to re-evaluate their stance. Once past the pre-contemplation stages, patients should be provided with the opportunity to utilize nicotine replacement therapy, pharmacotherapy, support groups, and continued education to maximize the chances of smoking cessation.
